# Diagnostic Accuracy of Smartphone-Based Patient-Initiated Store-and-Forward Teledermatology: A Cross-Sectional Direct-to-Specialist Study

**DOI:** 10.3390/diagnostics16172883

**Published:** 2026-09-07

**Authors:** Dominyka Stragyte, Gvidas Mikalauskas, Ugne Tiskeviciute, Katrina Gaidulevic, Renata Paukstaitiene, Kestutis Stasaitis, Skaidra Valiukevicienė

**Affiliations:** 1 Department of Skin and Venereal Diseases, Lithuanian University of Health Sciences, LT-44307 Kaunas, Lithuania; 2 Faculty of Medicine, Medical Academy, Lithuanian University of Health Sciences, LT-44307 Kaunas, Lithuania; 3 Department of Physics, Mathematics and Biophysics, Lithuanian University of Health Sciences, LT-44307 Kaunas, Lithuania; 4 Department of Emergency Medicine, Lithuanian University of Health Sciences, LT-44307 Kaunas, Lithuania

**Keywords:** smartphone-based teledermatology, patient-initiated teledermatology, direct-to-specialist, diagnostic accuracy

## Abstract

**Background:** Smartphone-based store-and-forward teledermatology (SAF-TD) has emerged as a potential approach to improving access to dermatological care. Patient-initiated SAF-TD systems, which enable direct image submission to dermatology specialists, represent an increasingly relevant but insufficiently studied model. However, diagnostic agreement remains insufficiently characterized in real-world, patient-initiated, direct-to-specialist systems in which adult patients independently submit conventional smartphone photographs and assessments are performed by dermatologists with different levels of clinical experience. **Methods:** A prospective cross-sectional, single-center study was conducted between February 2022 and September 2024, which included 83 patients. Patients independently submitted conventional smartphone photographs through a hospital patient portal. Diagnoses established remotely by an experienced and a beginner dermatologist were compared with face-to-face (FTF) diagnoses as the reference standard. Melanocytic lesions were defined as the positive category and non-melanocytic conditions as the negative category. Diagnostic agreement was assessed using Cohen’s kappa, and sensitivity and specificity were calculated with 95% confidence intervals (CIs). **Results:** Agreement between SAF-TD and FTF classification was 91.6% (95% CI, 83.6–95.9) for the experienced dermatologist (κ = 0.781; 95% CI, 0.628–0.934) and 90.4% (95% CI, 82.1–95.0) for the beginner dermatologist (κ = 0.760; 95% CI, 0.603–0.917), indicating substantial agreement. Sensitivity and specificity were 94.7% (95% CI, 75.4–99.1) and 90.6% (95% CI, 81.0–95.6), respectively, for the experienced dermatologist and 86.4% (95% CI, 66.7–95.3) and 91.8% (95% CI, 82.2–96.4), respectively, for the beginner dermatologist. **Conclusions:** Patient-initiated smartphone-based SAF-TD demonstrated substantial agreement with FTF binary classification by both dermatologists. These findings support its potential clinical use as a complementary method for initial remote dermatological assessment. However, the results require validation in larger, multicenter studies.

## 1. Introduction

Teledermatology (TD) has become an important component of modern digital healthcare, enabling remote evaluation, diagnosis, and management of dermatological conditions. Its diagnostic reliability compared with face-to-face (FTF) assessment has been extensively investigated. A recent systematic review and meta-analysis of 44 studies reported a pooled diagnostic agreement of 68.9% and a κ value of 0.67, indicating substantial agreement and supporting TD as a clinically useful diagnostic approach [1]. Importantly, agreement was higher when both TD and FTF assessments were performed by dermatologists rather than non-specialists (71% vs. 44%; κ = 0.69 vs. 0.52), highlighting the importance of specialist evaluation in remote dermatological care [1].

Several studies have evaluated patient-initiated TD. Pathipati and Ko demonstrated the feasibility of a direct-care TD service integrated into an academic medical center, whereas Green et al. confirmed the practicality of patient-initiated store-and-forward teledermatology (SAF-TD) in routine clinical practice [2,3]. However, these studies primarily addressed service feasibility, implementation, and access to specialist care and did not systematically evaluate diagnostic agreement against subsequent FTF dermatological assessment [2,3].

Other investigations have examined the clinical value of patient or caregiver-acquired smartphone images. O’Connor et al. demonstrated that parent-submitted smartphone photographs achieved an overall diagnostic concordance of 83% for pediatric skin conditions, increasing to 89% when image quality was considered adequate [4]. These findings supported the clinical utility of non-professionally acquired images; however, the study was restricted to a pediatric population and did not evaluate direct patient-to-specialist TD in adults [4].

Jiang et al. found that patient-submitted SAF-TD images were considered useful for clinical decision-making in 55.1% of evaluations and of sufficient quality in 62.2% [5]. This study highlighted the variability of patient-acquired photographs but focused on perceived image quality and usefulness rather than diagnostic agreement with subsequent FTF assessment [5]. Image quality was not quantitatively evaluated in the present study and was therefore considered a potential influencing factor rather than a measured outcome.

Smartphone-based TD has also been evaluated in specific clinical settings and populations. Shin et al. reported good diagnostic performance for smartphone-based consultations transmitted through a multimedia messaging service in the Korean military [6]. Although the study supported the clinical utility of smartphone images for common skin diseases, it was conducted within a structured military healthcare system rather than a routine patient-initiated, direct-to-specialist setting [6].

More recently, Fan et al. evaluated direct-to-patient mobile teledermoscopy and reported substantial agreement between remote and in-office diagnostic and management decisions [7]. Their findings demonstrated that patients could acquire clinically useful images outside healthcare facilities. However, the model relied on smartphone dermoscopic attachments, and most evaluated lesions were benign [7]. Consequently, it differed from routine patient-initiated SAF-TD based exclusively on conventional smartphone photographs without additional imaging equipment.

The studies most directly relevant to patient-initiated TD have addressed service feasibility, perceived image quality, selected pediatric or military populations, or teledermoscopy-assisted imaging [2,3,4,5,6,7]. However, they did not evaluate the specific combination of a real-world, patient-initiated, direct-to-specialist pathway; independent acquisition of conventional smartphone photographs by adult patients; subsequent FTF assessment of the same cases; and comparison of diagnostic agreement between dermatologists with different levels of clinical experience [2,3,4,5,6,7]. Consequently, the diagnostic performance of this specific care model remains insufficiently characterized.

In contrast to previous studies, the model evaluated in the present study combines three relatively understudied features: direct patient initiation through an institutional healthcare portal without prior referral or clinician triage; independent image acquisition using patients’ own smartphones without dermoscopic attachments or standardized imaging hardware; and diagnostic assessment by dermatologists with different levels of clinical experience. Early studies demonstrated that remote assessment of skin lesions, including melanocytic lesions, may serve as a viable alternative to face-to-face (FTF) consultation [8]. With advances in mobile technologies, the use of ubiquitous mobile optical sensor has further expanded the feasibility of TD in clinical practice [9]. Subsequent research has shown that patient-submitted images can provide clinically useful diagnostic information, including patient-initiated TD and mobile teledermoscopy models [7]. Studies have also demonstrated that such approaches can achieve acceptable diagnostic accuracy compared to in-person consultations, even in specific populations such as pediatric patients [4]. The increasing implementation of store-and-forward teledermatology (SAF-TD) systems has been supported by evidence demonstrating moderate to substantial diagnostic agreement with FTF consultations [1,10]. Advances in digital imaging and structured workflows have improved the reliability and clinical applicability of TD in routine practice [1,10]. Additionally, the quality of patient-submitted images has been identified as a critical factor influencing diagnostic performance [9]. Emerging technologies, including artificial intelligence, have also demonstrated potential to enhance mobile optical sensor-based dermatological assessment [11].

Importantly, patient-initiated and direct-care TD models have emerged as a significant development in healthcare delivery. These approaches allow patients to directly submit clinical images to dermatologists without prior triage and have been shown to be feasible in real-world clinical settings [2,3]. More recent studies focusing on direct-to-patient and patient-initiated systems have highlighted their potential to improve access to specialist care, optimize clinical workflows, and reduce delays in diagnosis [7]. However, while previous studies have primarily focused on feasibility and implementation of TD systems, robust evidence evaluating diagnostic accuracy and agreement in real-world mobile optical sensor-based settings without additional imaging devices remains limited. Therefore, this study aimed to evaluate the diagnostic accuracy and agreement of a patient-initiated mobile optical sensor-based SAF-TD system using standard clinical images and to compare its performance with FTF consultations across different lesion types and levels of clinician experience.

The rapid advancement of computer vision and machine learning (ML) in dermatology requires robust, reliable input from optical sensors. However, before deploying AI-driven diagnostic frameworks on uncalibrated, patient-initiated sensor streams, the baseline diagnostic viability of these ubiquitous optical sensors must be established. Therefore, this study aims to evaluate the diagnostic accuracy of patient-initiated mobile optical sensor data using human expert validation as the ground truth. Establishing this baseline performance across different lesion types is a critical prerequisite for the future development of image enhancement algorithms and ML models optimized for uncalibrated sensor data.

## 2. Materials and Methods

### 2.1. Study Design and Setting

This study was designed as a prospective, cross-sectional study evaluating diagnostic agreement in a real-world, smartphone-based, patient-initiated store-and-forward teledermatology (SAF-TD) model compared with face-to-face (FTF) consultation. The study was conducted at the Department of Skin and Venereal Diseases, Hospital of Lithuanian University of Health Sciences Kauno Klinikos (LSMUL KK), over a period from February 2022 and September 2024. No formal a priori sample size calculation was performed; all consecutive eligible patients who initiated a teleconsultation during the study period were invited to participate, and the precision of the resulting estimates is reported using 95% confidence intervals (CIs).

### 2.2. Study Population

Participants were adults who independently accessed the Kauno Klinikos Patient Portal (PP) and agreed to participate in the study. All participants provided informed consent prior to inclusion.

Inclusion criteria were:

1. Age ≥18 years;
2. Ability to use the PP and smartphone;
3. Submission of images meeting predefined quality requirements;
4. Willingness to attend a subsequent FTF dermatology consultation.

Exclusion criteria were:

1. Refusal to participate;
2. Inability to use the PP or smartphone;
3. Failure to submit digital images via the PP;
4. Conditions impairing the ability to provide informed consent.

During the study period, 90 patients completed a teleconsultation submission. Seven were excluded from the final analysis: one did not meet the adult age criterion, four did not attend the subsequent FTF consultation, and two submitted photographs that did not provide sufficient visual information for remote diagnostic assessment. Therefore, 83 adult patients with complete paired SAF-TD and FTF assessments were included in the final analysis.

### 2.3. Teledermatology Procedure

**Figure 1:**
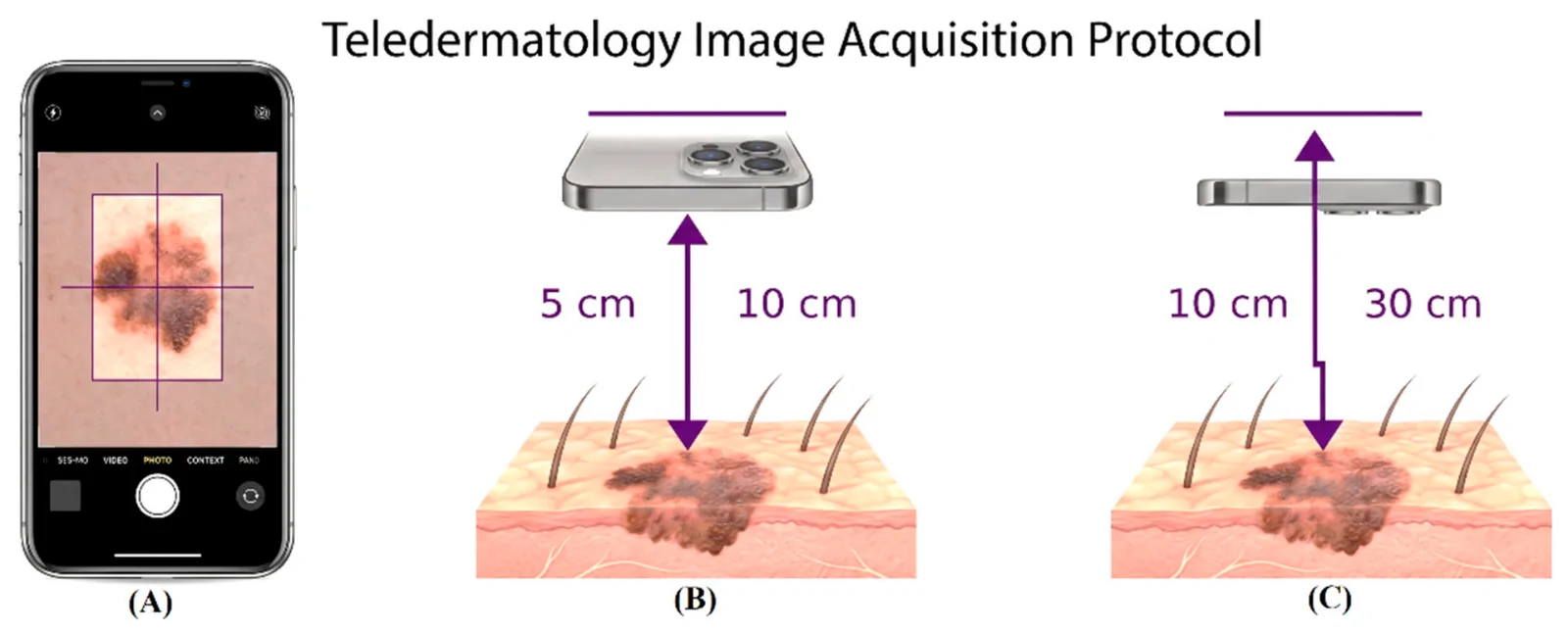
Standardized instructions for clinical image acquisition in smartphone-based patient-initiated teledermatology. The lesion should be positioned in the center of the frame (**A**), with both close-up images (approximately 5–10 cm) (**B**) and overview images (approximately 10–30 cm) (**C**) to ensure optimal diagnostic evaluation.

**Figure 2:**
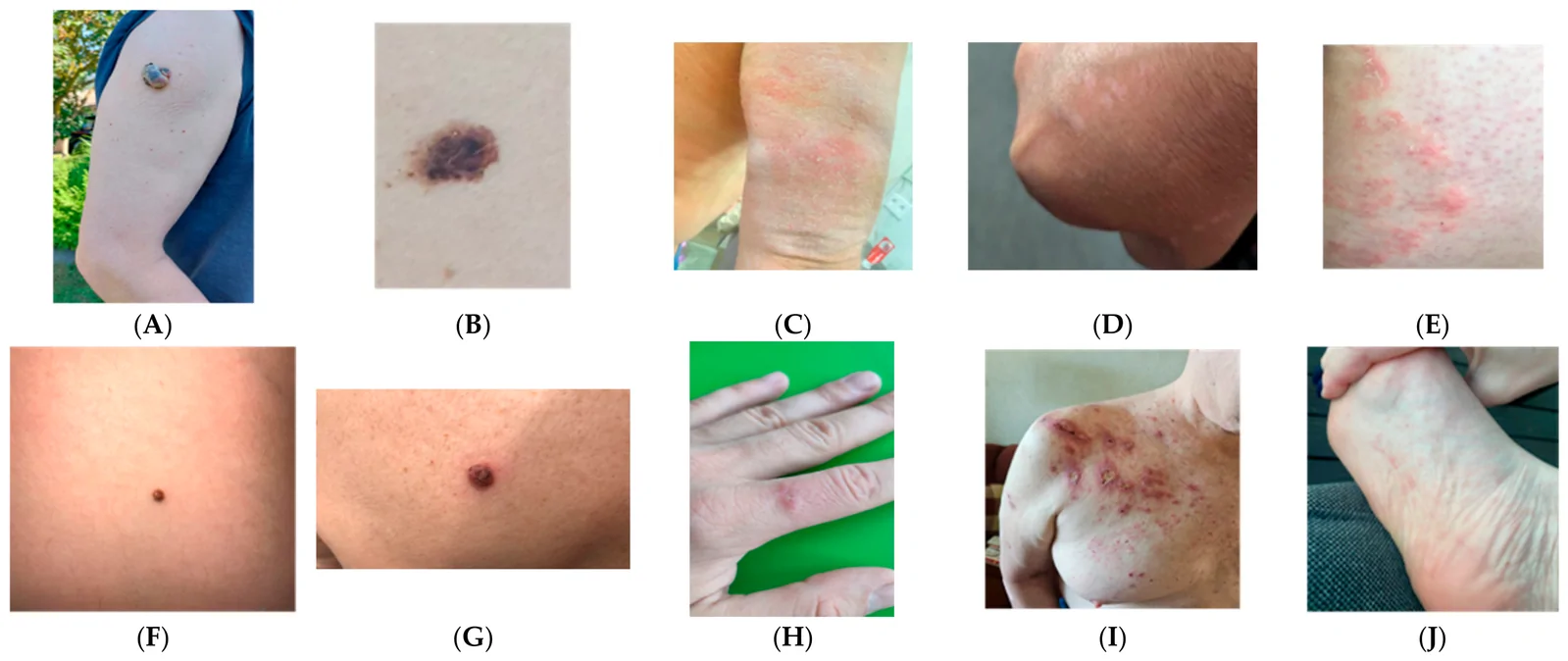
Representative patient-submitted clinical images used for SAF-TD, demonstrating examples of pigmented and non-pigmented skin lesions: (**A**,**B**,**F**,**G**) pigmented lesions and (**C**,**D**,**E**,**H**,**I**,**J**) non-pigmented skin conditions.

Participants independently captured digital images of their skin lesions or rashes using personal smartphone cameras according to standardized instructions provided within the PP system (Figure 1). No dermoscopic attachments or other specialized imaging devices were used. The images were uploaded through the secure PP platform. Standardized instructions for image acquisition were provided to patients through the PP. These included recommendations regarding adequate lighting (preferably natural light), appropriate distance from the lesion, and clear visualization of the affected area (Figure 1). Patients were allowed to submit one or more images per case (Figure 2).

Two submissions were considered insufficient for remote diagnostic assessment and were excluded. Patients were not asked to repeat image acquisition or resubmit photographs. Image quality was not assessed using a standardized scoring system; therefore, its association with diagnostic performance was not evaluated. The submitted images were evaluated using a SAF-TD approach. Each case was independently assessed by two dermatologists:

1. A beginner dermatologist with less than two years of clinical experience;
2. An experienced dermatologist with specialist-level expertise.

Both dermatologists provided their diagnoses based solely on the submitted images without access to additional clinical information or each other’s evaluations.

### 2.4. Face-to-Face Consultation

**Figure 3:**
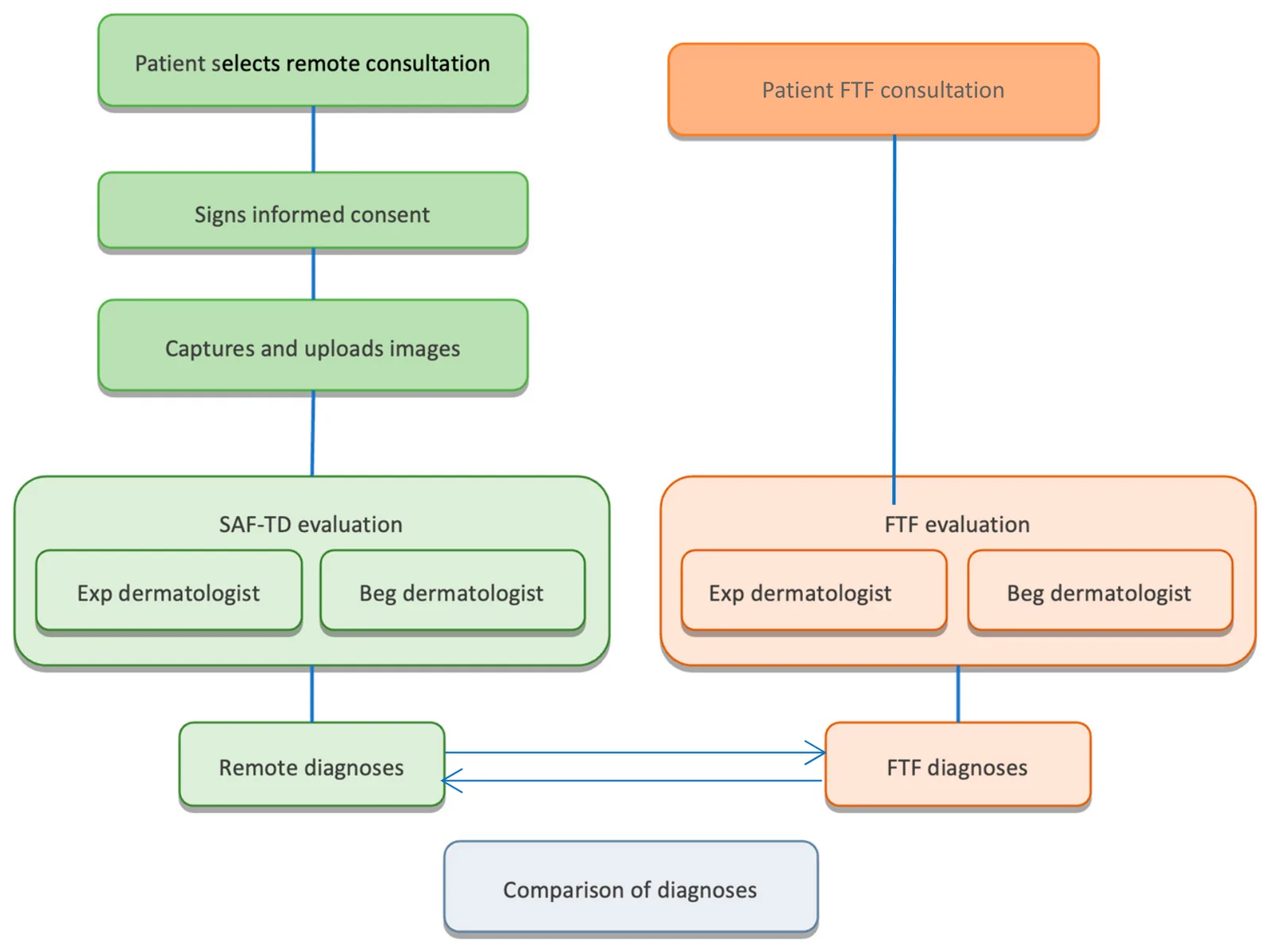
Workflow of patient-initiated SAF-TD and diagnostic comparison with FTF consultation. Patients independently initiated TD consultation via a smartphone-based PP application and provided informed consent. Clinical images were captured and uploaded by patients. The images were evaluated remotely using SAF-TD by both a beginner (Beg) dermatologist and an experienced (Exp) dermatologist. Subsequently, all patients underwent FTF consultation, where diagnoses were independently established by both dermatologists. Diagnostic outcomes from SAF-TD and FTF consultations were then compared.

Following the remote evaluation, all patients included in the final analysis underwent an in-person dermatological consultation at LSMUL Kauno Klinikos. The interval between image submission and the subsequent FTF consultation was recorded for each participant. The median interval was 4 days (IQR, 2–6 days), as reported in Table 1. During this FTF visit, both the beginner and experienced dermatologists independently performed clinical examinations and established their diagnoses under routine clinical conditions (Figure 3). The dermatologists were not formally blinded to their previous SAF-TD assessments. The FTF diagnoses established by each dermatologist served as the reference standard for that dermatologist’s remote assessment. For pigmented lesions, the FTF evaluation included dermoscopic examination as part of routine clinical practice. Histopathological examination was performed when clinically indicated, including for all lesions suspected of malignancy. Histopathological confirmation was available for melanoma (*n* = 2) and cutaneous carcinoma (*n* = 1). For inflammatory skin diseases, diagnoses were established clinically during the FTF consultation, and histopathological examination was not routinely performed. Histopathology was reserved for diagnostically uncertain or uncommon conditions and was available for one case of cutaneous scleroderma (*n* = 1).

### 2.5. Data Collection and Management

For each participant, anonymized data were collected, including demographic characteristics (age and sex), clinical diagnosis, and lesion characteristics. Each participant was assigned a unique study identification code to ensure confidentiality.

All data were stored in a secure electronic database and analyzed in an anonymized form to prevent identification of individual participants.

### 2.6. Outcome Measures

The primary outcome of the study was diagnostic agreement between SAF-TD and FTF consultation.

For subgroup analysis, lesions were classified as pigmented or non-pigmented. Pigmented lesions included melanoma and melanocytic nevi, while non-pigmented lesions comprised all other dermatological conditions, including both neoplastic and inflammatory diseases.

This classification is consistent with established dermatological diagnostic approaches, in which lesions are initially categorized as melanocytic (pigmented) or non-melanocytic (non-pigmented). Such differentiation represents a fundamental step in dermoscopic evaluation and clinical decision-making.

For the binary diagnostic performance analysis, pigmented lesions were treated as the positive category and non-pigmented skin conditions as the negative category. The corresponding FTF classification established by each dermatologist served as the reference standard for that dermatologist’s SAF-TD assessment.

Secondary outcomes included:

1. Agreement between beginner and experienced dermatologists;
2. Sensitivity and specificity of SAF-TD diagnoses;
3. Comparison of diagnostic accuracy between different lesion types.

### 2.7. Statistical Analysis

Statistical analysis was performed using Microsoft Excel and IBM SPSS Statistics software 32.0.0.

Diagnostic agreement was assessed using Cohen’s kappa coefficient (κ), interpreted according to the Landis and Koch criteria. Raw agreement, sensitivity, specificity, positive predictive value (PPV), and negative predictive value (NPV) were reported with 95% confidence intervals (CIs) calculated using the Wilson score method. The 95% CIs for Cohen’s κ were calculated using the asymptotic standard error method. All statistical tests were two-sided, and a *p*-value < 0.05 was considered statistically significant. An exploratory comparison of classification agreement between melanocytic and non-melanocytic cases was performed separately for each dermatologist using a two-sided Fisher’s exact test because of the small cell counts.

### 2.8. Ethical Considerations

The study was conducted in accordance with the Declaration of Helsinki. The study protocol was approved by the Kaunas Regional Biomedical Research Ethics Committee (approval No. BE-2-65, issued on 9 August 2022). Written informed consent was obtained from all participants prior to inclusion in the study. All data were anonymized to ensure participant confidentiality.

## 3. Results

A total of 83 patients (34 men and 49 women) were included in the study (Table 1). Based on the FTF diagnoses established by the experienced dermatologist, 19 cases (22.9%) were classified as melanocytic lesions and 64 cases (77.1%) as non-melanocytic skin conditions. The study population included a heterogeneous spectrum of dermatological conditions. The distribution of the individual FTF diagnoses is presented in Table 2.

Agreement between SAF-TD and FTF classification was 91.6% (76/83 cases; 95% CI, 83.6–95.9) for the experienced dermatologist and 90.4% (75/83 cases; 95% CI, 82.1–95.0) for the beginner dermatologist. The corresponding Cohen’s κ values were 0.781 (95% CI, 0.628–0.934; *p* < 0.001) and 0.760 (95% CI, 0.603–0.917; *p* < 0.001), respectively.

The underlying SAF-TD and FTF cross-tabulations are presented in Table 3. Using melanocytic lesions as the positive category and the corresponding FTF assessment as the reference standard, the experienced dermatologist achieved a sensitivity of 94.7% (95% CI, 75.4–99.1), specificity of 90.6% (95% CI, 81.0–95.6), PPV of 75.0% (95% CI, 55.1–88.0), and NPV of 98.3% (95% CI, 91.0–99.7). The corresponding estimates for the beginner dermatologist were 86.4% (95% CI, 66.7–95.3), 91.8% (95% CI, 82.2–96.4), 79.2% (95% CI, 59.5–90.8), and 94.9% (95% CI, 86.1–98.3), respectively. The diagnostic performance estimates are summarized in Table 4.

In an exploratory analysis, classification agreement did not differ significantly between melanocytic and non-melanocytic cases for either dermatologist. For the experienced dermatologist, agreement was 94.7% (18/19 cases) in the melanocytic group and 90.6% (58/64 cases) in the non-melanocytic group (two-sided Fisher’s exact test, *p* = 1.000). For the beginner dermatologist, the corresponding agreement was 86.4% (19/22 cases) and 91.8% (56/61 cases), respectively (two-sided Fisher’s exact test, *p* = 0.431). These findings should be interpreted cautiously because of the small subgroup sizes.

Interobserver agreement was evaluated at the level of individual diagnoses. During SAF-TD assessment, the two dermatologists assigned the same diagnosis in 80 of 83 cases (96.4%; 95% CI, 89.9–98.8), corresponding to almost perfect agreement (κ = 0.960; 95% CI, 0.915–1.000). During FTF assessment, the diagnoses agreed in 72 of 83 cases (86.7%; 95% CI, 77.8–92.4), also indicating almost perfect agreement (κ = 0.854; 95% CI, 0.774–0.933).

When exact individual diagnoses rather than the broader diagnostic categories were compared, SAF-TD and FTF diagnoses differed in 20 of 83 cases for the experienced dermatologist and 19 of 83 cases for the beginner dermatologist. Both histopathologically confirmed melanomas were correctly identified during SAF-TD by both dermatologists; however, the single histopathologically confirmed cutaneous carcinoma was not identified as malignant during either remote assessment.

**Figure 4:**
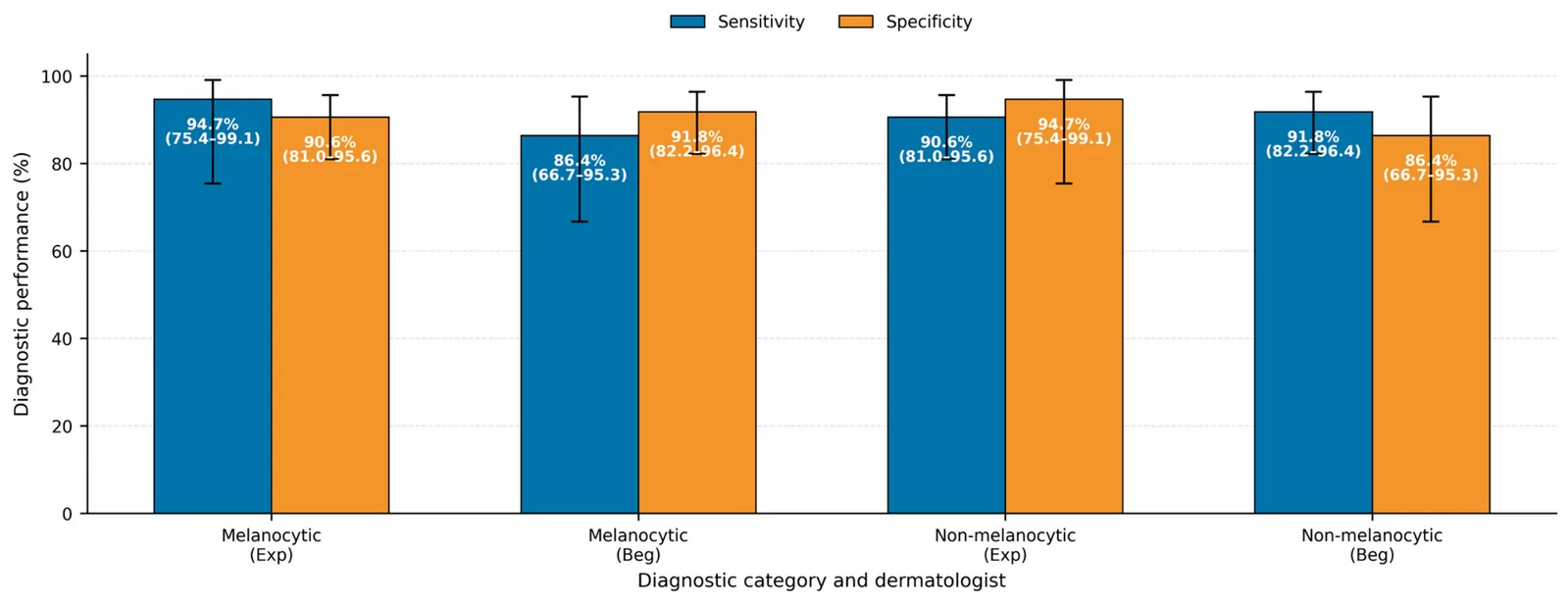
Sensitivity and specificity of SAF-TD compared to FTF consultation for pigmented and non-pigmented lesions, evaluated by beginner (Beg) and experienced (Exp) dermatologists.

Sensitivity and specificity estimates for melanocytic and non-melanocytic cases, evaluated separately by the experienced and beginner dermatologists, are presented with 95% confidence intervals in Figure 4.

## 4. Discussion

This study demonstrates that smartphone-based SAF-TD, implemented as a patient-initiated, direct-to-specialist model, achieves substantial diagnostic agreement with FTF consultations. To the best of our knowledge, this is one of the few studies evaluating diagnostic agreement in a real-world, patient-initiated, direct-to-specialist TD setting in which adult patients independently submit conventional smartphone photographs without dermoscopic attachments [2,3,4,5,6,7]. Agreement with the corresponding FTF binary classification was 91.6% for the experienced dermatologist (κ = 0.781) and 90.4% for the beginner dermatologist (κ = 0.760), indicating substantial agreement according to the Landis and Koch criteria. These findings are consistent with the broader evidence supporting the diagnostic reliability of TD compared with in-person assessment [1,10]. However, direct comparisons should be interpreted cautiously because previous studies differed in their populations, diagnostic categories, image-acquisition methods, and reference standards.

The exploratory analysis did not identify statistically significant differences in classification agreement between melanocytic and non-melanocytic cases for either dermatologist. The small number of melanocytic cases limited the statistical power of these comparisons. Therefore, the absence of statistical significance should not be interpreted as evidence of equivalent diagnostic performance between the groups. Differences in lesion morphology and image characteristics between melanocytic and non-melanocytic cases could theoretically influence diagnostic assessment based on digital images; however, this possibility was not directly evaluated in the present study and should therefore be regarded as a hypothesis requiring further investigation.

The analysis of exact diagnostic discordances provides important clinical context. When exact individual diagnoses rather than the broader binary categories were compared, SAF-TD and FTF diagnoses differed in 20 of 83 cases for the experienced dermatologist and 19 of 83 cases for the beginner dermatologist. The most frequent discordance was a SAF-TD diagnosis of melanocytic nevus followed by an FTF diagnosis of seborrheic keratosis, occurring in four cases for each dermatologist. Differentiation between melanocytic lesions and seborrheic keratoses may be challenging in image-based assessment; a previous study evaluating a smartphone-based AI application using dermoscopic images reported lower sensitivity for seborrheic keratosis classification than for melanoma and melanocytic nevus classification [11]. However, that study used dermoscopic images and an automated algorithm and is therefore not directly comparable with the present patient-initiated SAF-TD model. Both histopathologically confirmed melanomas were correctly identified during SAF-TD by both dermatologists, whereas the single histopathologically confirmed cutaneous carcinoma was not identified as malignant during either remote assessment. These findings support the complementary role of SAF-TD in initial dermatological assessment and highlight the importance of FTF evaluation when malignancy is suspected, or the remote diagnosis remains uncertain.

The findings of this study are consistent with earlier research demonstrating the feasibility of patient-initiated and direct-care TD models. Previous studies have shown that systems in which patients directly submit images to dermatologists without preselection or referral are feasible in clinical practice [2,3,7]. O’Connor et al. demonstrated that caregiver-acquired smartphone photographs can provide clinically useful diagnostic information, although their study was restricted to a pediatric population [4]. Shin et al. reported good diagnostic performance in a structured military healthcare setting [6], whereas Fan et al. demonstrated the clinical utility of patient-acquired mobile teledermoscopy [7]. These studies support the broader clinical value of remotely acquired images but differ from the present adult, patient-initiated model using conventional smartphone photographs without dermoscopic attachments.

However, an important distinction of the present study lies in its focus on diagnostic agreement rather than feasibility. While previous studies have primarily evaluated implementation and usability of patient-initiated TD systems, the present study extends the existing evidence by providing a quantitative evaluation of diagnostic agreement compared with subsequent FTF consultations. Importantly, this study reflects a real-world scenario using standard smartphone images without additional dermoscopic equipment, an aspect that has been insufficiently addressed in previous research.

Unlike clinician-assisted TD, in which image acquisition may be performed or supervised by trained personnel, patient-initiated SAF-TD depends on patients’ ability to capture and submit clinically informative photographs independently. This approach may improve direct access to specialist assessment but introduces variability in lighting, focus, image distance, anatomical coverage, and adherence to image-acquisition instructions. Jiang et al. found that patient-submitted SAF-TD images were considered useful for clinical decision-making in 55.1% of evaluations and of sufficient quality in 62.2%, illustrating the variability of patient-acquired photographs [5]. Previous research has similarly demonstrated that image quality may affect the ability to establish a correct remote diagnosis [9].

In contrast to clinician-assisted or dermoscopy-assisted imaging models [7,8], the present study reflects routine clinical practice where patients rely solely on smartphone photography. Although this approach increases practical accessibility, variability in patient-acquired photographs may influence diagnostic performance. In the present study, patients received standardized image-acquisition instructions; however, image quality, anatomical location, lighting conditions, and adherence to individual instructions were not assessed quantitatively. Therefore, their independent effects on diagnostic performance could not be determined.

Interobserver agreement at the level of individual diagnoses was high. This may partly reflect the inclusion of many common and clinically recognizable dermatological conditions, which may have facilitated consistent diagnostic assessment. However, the observed agreement should be interpreted cautiously because it was obtained in a relatively small, single-center cohort. Furthermore, high interobserver agreement does not necessarily indicate equivalent diagnostic accuracy across different levels of clinical experience. Further studies involving larger and more diagnostically diverse cohorts are needed to evaluate the reproducibility of these findings. The higher agreement reported when both remote and FTF assessments are performed by dermatologists rather than non-specialists further supports the importance of specialist involvement in TD pathways [1].

From a clinical perspective, these findings support the potential integration of patient-initiated SAF-TD into routine dermatological workflows as a complementary pathway for initial specialist assessment and triage. Successful implementation requires clear image-acquisition instructions, secure communication through the patient portal, and a defined pathway to FTF consultation when remote assessment is inconclusive.

The practical value of teledermatology extends beyond diagnostic agreement. Tatu described how TD supported access to dermatological assessment during the COVID-19 pandemic despite repeated departmental relocation and disruption of conventional clinical services [12]. This real-world experience highlights the adaptability of TD and its potential to support continuity of care when routine FTF services are limited.

By enabling direct access to specialist evaluation, such models have the potential to improve healthcare accessibility and maintain continuity of dermatological care [2,3,7,12]. However, patient-initiated SAF-TD should be considered a complement to, rather than a replacement for, FTF assessment, particularly when the remote diagnosis is uncertain or malignancy is suspected.

Several limitations of this study should be acknowledged. First, the relatively small sample size—with only 24 pigmented lesions—and the single-center design limit the precision and generalizability of the estimates, as reflected by the wide confidence intervals; no formal a priori sample size calculation was performed. Second, participation required smartphone access, sufficient digital literacy to use the patient portal, successful submission of photographs considered suitable for remote assessment, and attendance at the subsequent FTF consultation. These requirements may have introduced selection bias and may limit the applicability of the findings to patients with limited access to or experience with digital healthcare technologies. Third, the dermatologists who established the FTF reference diagnoses were the same physicians who had previously evaluated the images remotely and were not formally blinded to their own SAF-TD assessments. Given the median interval of 4 days between the two assessments, recall of the previous remote diagnosis cannot be excluded and may have increased the observed agreement. Fourth, the FTF clinical diagnosis served as the reference standard, and histopathological confirmation was available only when clinically indicated; therefore, misclassification of the reference standard cannot be excluded. Fifth, the primary diagnostic performance analysis was based on broad binary classification into melanocytic and non-melanocytic categories rather than exact individual diagnoses. The non-melanocytic category included a heterogeneous range of neoplastic, inflammatory, and other dermatological conditions. Finally, two submissions that did not provide sufficient visual information for remote diagnostic assessment were excluded, and the patients were not asked to resubmit photographs. Image quality was not assessed using a standardized scoring system; therefore, its association with diagnostic performance could not be evaluated, and the reported estimates apply only to submissions considered suitable for remote assessment. No dermoscopic imaging was available for the SAF-TD assessments. These factors should be considered when interpreting the diagnostic performance and generalizability of the findings.

Future research should focus on multicenter studies with larger and more diagnostically diverse cohorts and standardized imaging protocols to further validate these findings. Overall, this study demonstrates that patient-initiated smartphone-based SAF-TD can achieve substantial diagnostic agreement with FTF consultations under real-world conditions, supporting its role as a potentially useful complement to conventional dermatological care.

## 5. Conclusions

This study demonstrated substantial agreement between patient-initiated SAF-TD based on patient-acquired smartphone photographs and FTF classification into pigmented and non-pigmented categories. The findings support the potential clinical use of this approach as a complementary method for initial remote dermatological assessment rather than a replacement for FTF consultation. However, given the relatively small, single-center cohort, the results should be interpreted cautiously and validated in larger, multicenter studies.

## Figures and Tables

**Table 1:** Demographic and Clinical Characteristics of the Study Population.

Characteristic	Value
Total number of participants, *n*	83
Age, years, mean ± SD (range)	38.9 ± 14.0 (18–80)
Female, *n* (%)	49 (59.0)
Male, *n* (%)	34 (41.0)
Median interval between image submission and FTF consultation, days (IQR)	4 (2–6)
Pigmented lesions (SAF-TD), *n* (%)	24 (28.9)
Non-pigmented lesions (SAF-TD), *n* (%)	59 (71.1)
Pigmented lesions (FTF), *n* (%)	19 (22.9)
Non-pigmented lesions (FTF), *n* (%)	64 (77.1)
Histopathologically confirmed diagnosis, *n*	4

FTF, face-to-face; IQR, interquartile range; SAF-TD, store-and-forward teledermatology; SD, standard deviation.

**Table 2:** Distribution of face-to-face diagnoses established by the experienced dermatologist.

Diagnostic Category	FTF Diagnosis	*n* (%)
Melanocytic lesions	Subtotal	19 (22.9)
	Melanoma *	2 (2.4)
	Melanocytic nevus	17 (20.5)
Non-melanocytic skin conditions	Subtotal	64 (77.1)
	Seborrheic keratosis	9 (10.8)
	Basal cell carcinoma *	1 (1.2)
	Actinic keratosis	2 (2.4)
	Dermatofibroma	5 (6.0)
	Haemangioma	4 (4.8)
	Viral wart and molluscum contagiosum	3 (3.6)
	Lipoma and epidermoid cyst	3 (3.6)
	Pyogenic granuloma	1 (1.2)
	Allergic contact dermatitis	12 (14.5)
	Irritant contact dermatitis	3 (3.6)
	Atopic dermatitis	3 (3.6)
	Psoriasis	3 (3.6)
	Rosacea	1 (1.2)
	Seborrheic dermatitis	1 (1.2)
	Folliculitis	1 (1.2)
	Scabies and insect-bite reaction	4 (4.8)
	Scleroderma *	1 (1.2)
	Onychomycosis	2 (2.4)
	Leg ulcer	1 (1.2)
	Hematoma	1 (1.2)
	Pityriasis versicolor	1 (1.2)
	Erythema migrans	1 (1.2)
	Herpes zoster	1 (1.2)
Total		83 (100.0)

* Histopathologically confirmed diagnosis.

**Table 3:** Cross-tabulation of SAF-TD and FTF classification into melanocytic and non-melanocytic categories.

(A) Experienced Dermatologist			
**FTF classification**	**SAF-TD: melanocytic**	**SAF-TD: non-melanocytic**	**Total**
Melanocytic	18 (TP)	1 (FN)	19
Non-melanocytic	6 (FP)	58 (TN)	64
Total	24	59	83
(**B**) **Beginner Dermatologist**			
**FTF classification**	**SAF-TD: melanocytic**	**SAF-TD: non-melanocytic**	**Total**
Melanocytic	19 (TP)	3 (FN)	22
Non-melanocytic	5 (FP)	56 (TN)	61
Total	24	59	83

Melanocytic lesions were treated as the positive category, and the corresponding FTF assessment of each dermatologist was used as the reference standard. FN, false negative; FP, false positive; FTF, face-to-face; SAF-TD, store-and-forward teledermatology; TN, true negative; TP, true positive.

**Table 4:** Diagnostic performance of SAF-TD classification compared with the corresponding FTF assessment.

Measure	Experienced Dermatologist	Beginner Dermatologist
Agreement, *n*/N (%)	76/83 (91.6)	75/83 (90.4)
Agreement, % (95% CI)	91.6 (83.6–95.9)	90.4 (82.1–95.0)
Cohen’s κ (95% CI)	0.781 (0.628–0.934)	0.760 (0.603–0.917)
*p*-value for κ	<0.001	<0.001
Sensitivity, % (95% CI)	94.7 (75.4–99.1)	86.4 (66.7–95.3)
Specificity, % (95% CI)	90.6 (81.0–95.6)	91.8 (82.2–96.4)
PPV, % (95% CI)	75.0 (55.1–88.0)	79.2 (59.5–90.8)
NPV, % (95% CI)	98.3 (91.0–99.7)	94.9 (86.1–98.3)

Melanocytic lesions were treated as the positive category, and the corresponding FTF assessment of each dermatologist was used as the reference standard. N, total number of cases; n, number of agreements; CI, confidence interval; FTF, face-to-face; NPV, negative predictive value; PPV, positive predictive value; SAF-TD, store-and-forward teledermatology.

## Data Availability

The data will be made available by the corresponding author on request.

## Abbreviations

The following abbreviations are used in this manuscript:

SAF-TD	Store-and-forward teledermatology
FTF	Face-to-face consultation
TD	Teledermatology
PP	Kauno Klinikos Patient Portal

## References

[1] Bourkas A.N., Barone N., Bourkas M.E.C., Mannarino M., Fraser R.D.J., Lorincz A., Wang S.C., Ramirez-GarciaLuna J.L. (2023). Diagnostic reliability in teledermatology: A systematic review and meta-analysis. BMJ Open.

[2] Pathipati A.S., Ko J.M. (2016). Implementation and evaluation of Stanford Health Care direct-care teledermatology program. Telemed. J. e-Health.

[3] Green C.B., Chiang C., Plovanich M.E. (2016). Patient-initiated store-and-forward teledermatology: A single practice experience. Telemed. J. e-Health.

[4] O’Connor D.M., Jew O.S., Perman M.J., Castelo-Soccio L.A., Winston F.K., McMahon P.J. (2017). Diagnostic accuracy of pediatric teledermatology using parent-submitted photographs: A randomized clinical trial. JAMA Dermatol..

[5] Jiang S.W., Flynn M.S., Kwock J.T., Liu B., Quow K., Blanchard S.K., Breglio K.F., Fresco A., O’Brien Jamison M., Lesesky E. (2022). Quality and Perceived Usefulness of Patient-Submitted Store-and-Forward Teledermatology Images. JAMA Dermatol..

[6] Shin H., Kim D.H., Ryu H.H., Yoon S.Y., Jo S.J. (2014). Teledermatology consultation using a smartphone multimedia messaging service for common skin diseases in the Korean army: A clinical evaluation of its diagnostic accuracy. J. Telemed. Telecare.

[7] Fan W., Mattson G., Twigg A. (2024). Direct-to-patient mobile teledermoscopy: A prospective observational study. JMIR Dermatol..

[8] Lamel S.A., Haldeman K.M., Ely H., Kovarik C.L., Pak H., Armstrong A.W. (2012). Application of mobile teledermatology for skin cancer screening. J. Am. Acad. Dermatol..

[9] Weingast J., Scheibböck C., Wurm E.M., Ranharter E., Porkert S., Dreiseitl S., Posch C., Binder M. (2013). A prospective study of mobile phones for dermatology in a clinical setting. J. Telemed. Telecare.

[10] Bastola M., Locatis C., Fontelo P. (2021). Diagnostic reliability of in-person versus remote dermatology: A meta-analysis. Telemed. J. e-Health.

[11] Liutkus J., Kriukas A., Stragytė D., Mazeika E., Raudonis V., Galetzka W., Stang A., Valiukeviciene S. (2023). Accuracy of a smartphone-based artificial intelligence application for classification of melanomas, melanocytic nevi, and seborrheic keratoses. Diagnostics.

[12] Tatu A.L. (2024). Teledermatology Practice in a Department that Was Relocated Multiple Times during the COVID-19 Pandemic. Clin. Cosmet. Investig. Dermatol..

